# Selinexor in Combination with Decitabine Attenuates Ovarian Cancer in Mice

**DOI:** 10.3390/cancers15184541

**Published:** 2023-09-13

**Authors:** Patrick J. Stiff, Swati Mehrotra, Ronald K. Potkul, Swarnali Banerjee, Christopher Walker, Maureen L. Drakes

**Affiliations:** 1 Cardinal Bernardin Cancer Center, Department of Medicine, Loyola University Chicago, Maywood, IL 60153, USA; 2 Edward Hines Junior VA Hospital, Hines, IL 60141, USA; 3 Department of Obstetrics and Gynecology, Loyola University Chicago, Maywood, IL 60153, USA; 4 Department of Mathematics and Statistics, Loyola University Chicago, Chicago, IL 60660, USA; 5 Karyopharm Therapeutics, Newton, MA 02459, USA

**Keywords:** ovarian cancer, decitabine, selinexor, combination treatment, mouse model, disease improvement, anti-tumor response

## Abstract

**Simple Summary:**

Ovarian cancer is frequently discovered in the later stages. Current treatment for advanced disease ovarian cancer is inadequate and survival is poor. Our goal was to investigate how treatment with targeted therapy decitabine and/or selinexor could improve ovarian cancer in mice, when compared with experimental controls. Mice were administered ovarian cancer cells. About one week later, they were administered experimental treatment decitabine followed by selinexor. Disease outcome or improvement with single or combination treatment was studied by analyzing pathology changes in several organs to determine tumor spread and in biological experiments conducted on cells obtained from mice to investigate how treated mice developed anti-tumor immune responses to fight ovarian cancer. This study is of great significance as it may lead to new clinical trials with combination decitabine and selinexor and potentially to future therapies with these agents, which may prolong the life of women diagnosed with ovarian cancer.

**Abstract:**

Background. High-grade serous ovarian cancer is a lethal gynecologic disease. Conventional therapies, such as platinum-based chemotherapy, are rendered inadequate for disease management as most advanced disease patients develop resistance to this therapy and soon relapse, leading to poor prognosis. Novel immunotherapy and targeted therapy are currently under investigation as treatment options for ovarian cancer, but so far with little success. Epigenetic changes, such as aberrant DNA methylation, have been reported in resistance to platinum-based therapy. Decitabine is a hypomethylating agent which is effective against platinum-resistant disease and also exhibits several anti-tumor immune functions. Selinexor is a selective inhibitor of nuclear protein export. It restored platinum sensitivity in patient-derived ovarian cancer cell lines and is currently in clinical trials for the treatment of platinum-resistant ovarian cancer. We hypothesized that these two agents used in combination could elicit more potent anti-tumor immune responses in vivo than either agent used alone. Methods. These studies were designed to investigate the efficacy of these two agents used in combination to treat ovarian cancer by assessing murine models for changes in disease pathology and in anti-tumor responses. Results. Decitabine priming followed by selinexor treatment significantly limited ascites formation and tumor size. This combination of agents also promoted T cell effector function as measured by granzyme B secretion. Treatment of mice with decitabine and selinexor led to the significant release of a broader range of macrophage and T cell cytokines and chemokines above control PBS and vehicle and above decitabine or selinexor treatment alone. Conclusions. These results reveal crucial information for the design of clinical trials which may advance therapy outcomes in ovarian cancer.

## 1. Introduction

Ovarian cancer is the deadliest gynecological cancer [1]. In the US in 2023, approximately 19,710 new cases of ovarian cancer will be detected and about 13,270 women will die due to this disease [2]. This disease is initially responsive to chemotherapy using platinum-based regimens, but platinum-resistant disease typically develops early in treatment. Additional courses of platinum-based chemotherapy are of little benefit for these platinum-resistant patients and most of these patients soon die of disease. A smaller group of patients who relapse may be platinum-sensitive and will respond favorably to additional regimens of platinum-based chemotherapy and enjoy much longer survival [3,4,5,6].

Over the last three decades, there has been little improvement in the survival of patients with ovarian cancer [3,7]. This underscores the urgency of providing adequate management strategies and novel treatments for those afflicted with this disease [4,8]. In this effort, novel therapies, such as immune checkpoint inhibitors including anti-programmed death-1 (anti-PD-1) antibodies, have been investigated in ovarian cancer clinical trials, but these immunotherapy agents only give marginal improvements to patient outcome [9,10,11,12]. Poor response to such novel therapy may be due to several existing and emerging resistance mechanisms [13,14], and we urgently need to develop treatment options to combat several of these mechanisms together in patients to prevent disease progression and significantly impact survival in ovarian cancer. The studies described here focused on the combined action of two anti-cancer agents which can potentially significantly impact disease mechanisms in ovarian cancer.

Epigenetic changes can induce initiation and progression of cancers and resistance to therapies [15]. Aberrant DNA methylation has been implicated in resistance to platinum-based therapy in ovarian cancer [16,17,18]. Gene silencing caused by DNA hypermethylation can be reversed by DNA methyltransferase (DNMT) inhibitors, such as 5-aza-2′-deoxycytidine (decitabine) [17]. Decitabine is a hypomethylating agent that is FDA approved for the management of hematologic malignancies, such as myelodysplastic syndromes (MDS) [19] and acute myeloid leukemia (AML) [20,21], diseases in which many of these patients have aberrant DNA methylation with mutations in a several genes [22].

In ovarian cancer, reversal of drug resistance to platinum-based drugs is a useful therapeutic strategy [16,18]. Agents such as decitabine can upregulate the expression of hypermethylation-silenced tumor suppressor genes and increase the sensitivity of ovarian cancer cells to chemotherapy [17,23]. The use of decitabine in ovarian cancer clinical trials showed some promise. For example, a clinical trial with 17 ovarian cancer patients showed that decitabine used before carboplatin induced an objective response rate (ORR) in 35% of patients, with a progression-free survival (PFS) of 10.2 months [16]. Nine patients were progression-free at 6 months [16]. Other reports demonstrated that low dose decitabine with paclitaxel or carboplatin showed some benefit in patients with recurrent ovarian cancer [24].

Recent studies in BR5FVR-Akt ovarian cancer cells also suggest that decitabine can alter the expressions of several immune-related genes. After treatment of these cells with control DMSO or decitabine, the most upregulated gene ontology signature with decitabine treatment was the “immune system process” [25]. Additionally, in some reports, strong immunomodulatory effects have been identified with the use of decitabine treatment, including T cell infiltration in the tumor and priming of the tumor to make other agents, such as immune checkpoint blockers, a more potent treatment for cancer [26,27,28]. With this knowledge, we postulated that decitabine may be a useful anti-cancer agent as well as an effective anti-tumor agent to prime anti-tumor immunity in ovarian cancer.

Tumor suppressor proteins (TSP) prevent malignant transformation and are often mutated in cancers. The function of many tumor suppressor proteins is dependent on their localization in the nucleus [29,30]. Increased nuclear export of TSPs to the cytoplasm is a mechanism utilized by some cancer cells to functionally inactivate critical TSPs. Exportin 1 (XPO1) mediates the nuclear transport of over 200 mammalian cargo proteins, including TSPs, such as p73, p21, p53 and BRCA1, the latter two being important in ovarian cancer initiation, cell cycle progression and chemo-resistance. Upregulation of exportin-1 shuttles p53 and other proteins out of the nucleus. Increased expression of the XPO1 correlates with lower survival and with platinum resistance in ovarian cancer and other cancers [31,32].

Selinexor (KPT-330) is a selective inhibitor of nuclear export (SINE). It is a highly specific, slowly reversible covalent inhibitor of XPO1. It is FDA approved for use in adult patients with relapsed or refractory multiple myeloma [33] and has received accelerated approval in patients with relapsed or refractory diffuse large B-cell lymphoma (DLBCL) [34]. It is also used in clinical trials for endometrial cancer, ovarian and other gynecological malignancies, as well as for non-small cell lung cancer (NSCLC), colorectal cancer (CRC) and glioblastoma multiforme (GBM) [24,35,36,37,38]. In a clinical study evaluating selinexor treatment in 66 heavily pre-treated ovarian cancer patients with recurrent disease, the disease control rate (DCR) was 30%, which included a partial response of 8%. The median progression-free survival was 2.6 months, and the median overall survival was 1.3 months [37]. In pre-clinical studies, selinexor treatment reduced tumor burden in several xenograft and syngeneic mouse models [39,40]. Additionally, inhibition of XPO1 with selinexor restored platinum sensitivity in patient-derived ovarian cancer cell lines [32]. Both decitabine and selinexor have shown some effectiveness against platinum-resistant disease and can also potentiate anti-tumor immunity. We undertook a study to determine whether these agents, when used as a novel combination, could exceed the performance of either single therapy and be more effective in reducing disease in an ovarian cancer mouse model. These studies investigated the potency of single and combination regimens of decitabine and selinexor in the treatment of ovarian cancer in an immunocompetent ID8 syngeneic mouse model [41] using female C57BL/6 mice. The findings of this study demonstrate potent treatment efficacy of decitabine and selinexor treatment to limit ascites development and the size of tumors. This disease improvement was accompanied with an increased capacity of CD8+ T cells to secrete cytotoxic molecule granzyme B. Furthermore, treatment of mice with decitabine and selinexor combination led to the significant release of a broader range of macrophage and T cell cytokines and chemokines than what was observed for decitabine or selinexor treatment alone. The outcome of this pre-clinical study provides a rationale for the use of these two agents together in clinical trials for advanced disease ovarian cancer patients.

## 2. Methods

### 2.1. Culture of Tumor Cells and Induction of Ovarian Cancer in Mice

Mouse studies were performed with IACUC protocol approval and under the guidelines of treatments of animals at Loyola University Chicago, Maywood, IL, USA. Mouse ID8-red fluorescent protein (ID8-RFP) tumor cells were cultured in 75 cm^3^ flasks in Dulbecco’s Modification of Eagle’s Medium with 1 g/L glucose, L-glutamine and sodium pyruvate (DMEM 1×; Corning, VA, USA) supplemented with 1% insulin, transferrin, selenium (Gibco, Grand Island, NY, USA; 41400-045), 4% fetal bovine serum (FBS; Cytiva Hyclone SH 30910.03, South Logan, UT, USA) and 1% penicillin/streptomycin (Gibco, Grand Island, NY, US) in a 5% humidified CO_2_ incubator (3110 WJ Forma Series II, Marietta, OH, USA). Tumor cells were removed from the surface of flasks and split at a 1:3 ratio daily for one week, until sufficient cells were grown up in several flasks and recovered for use. The pooled ID8-RFP cells were washed in 1X phosphate buffered saline (PBS; Lonza BioWhittaker, Walkersville, MD, USA) with 2% FBS three times and given one final wash in PBS before administration to mice. To induce disease, 8- to 10-week old C57BL/6 (B6) mice (Jackson laboratories, Bar Harbor, ME, USA) were administered ID8-RFP cells at a dose of 1–1.5 × 10^6^ cells/mouse by intraperitoneal (I.P.) injection at day 0, using a fixed dose for all mice in the same experiment. All mice were fed an alfalfa-free diet to reduce auto-fluorescence in IVIS Spectrum (PerkinElmer, Inc. Waltham, MA, USA) imaging.

### 2.2. In Vivo Treatment of Mice

**Figure 1:**
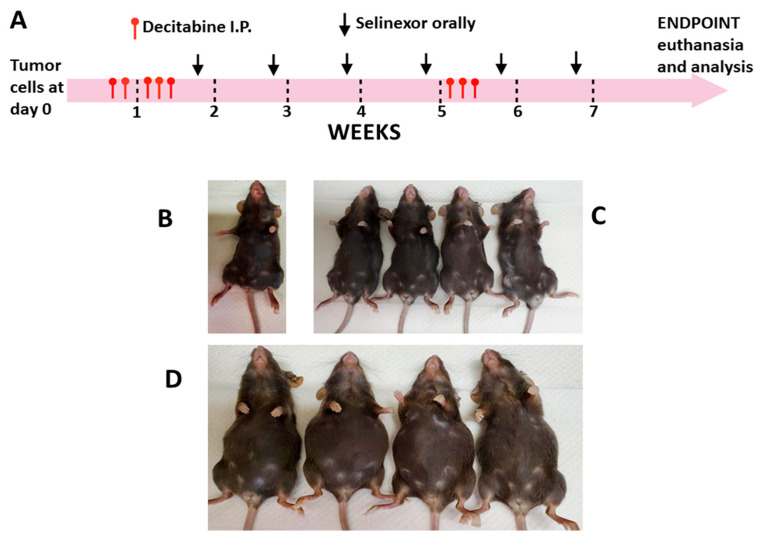
Representative schema for administration of treatment agents to mice. Mice were administered ID8-RFP tumor cells at day 0. Decitabine (1.25 mg/kg each dose or control PBS) and/or selinexor (10 mg/kg or control vehicle) was administered as single or in combination treatment at time points as indicated. Selinexor was reconstituted in vehicle consisting of 0.6% Plasdone PVP K-29/32 and 0.6% Poloxamer Pluronic F-68 in sterile distilled water. Panel (**A**) describes the basic regimen for treatment of mice. Panels (**B**–**D**) show the morphology of mice at the time of euthanasia. Mice were administered tumor cells followed by combination treatments. In this study, mice were euthanized on day 66 post-tumor cell inoculation. Panels show contrasting sizes for the abdomens in different groups. Panel (**B**) shows a normal unmanipulated mouse. Representative mice for treatment outcome with combination decitabine and selinexor (Panel (**C**), flat abdomens) and for PBS and vehicle controls (panel (**D**), enlarged abdomens) are shown. Results are representative of observations of two separate experiments. Enlarged abdomens were also found in single control PBS or control vehicle mice.

Mice were ear tagged, weighed and monitored before any treatments were administered and at frequent intervals thereafter, until the end of the study. Mice were randomized into groups of five in each cage for treatments. Decitabine (Selleck chemicals, Houston, TX, USA; S1200) was diluted in PBS and administered I.P. diluted in sterile PBS (control). Treatment started on day 6 after tumor cell inoculation and 5 doses were given over 7 days. There was cessation of decitabine treatment for about 4 weeks, then mice were given 3 more doses (1.25 mg/kg each dose) for a total of 8 doses for each mouse (Figure 1A).

Selinexor (KPT-330; Karyopharm Therapeutics, Newton, MA, obtained under Materials Transfer Agreement; MTA) was reconstituted in manufacturer supplied vehicle (0.6% Plasdone PVP K-29/32 and 0.6% Poloxamer Pluronic F-68 in sterile distilled water) and given orally starting at day 14 post-tumor cell inoculation, administering 1 dose weekly (10 mg/kg) for 5 or 6 weeks (5 or 6 doses total/mouse) (Figure 1A). All mice treated with selinexor within the same study received the same number of doses of selinexor. Six groups of mice were set up for treatment as follows: decitabine, PBS, selinexor, vehicle, decitabine and selinexor and PBS and vehicle. Normal unmanipulated B6 mice were also set up as additional study controls.

### 2.3. Monitoring of Mice and Ascites Recovery

Mice were monitored and observed at least 4 times weekly from day 0 of tumor cell administration over the duration of the study. The primary end point was determined as when the PBS or the vehicle control mice had significantly extended abdomens due to ascites formation but were not yet lethargic. At this time, corresponding experimental and control groups of mice were weighed and then euthanized for analysis of cells and tissues (Figure 1B). For endpoint studies, an equal number of control and of experimental mice were euthanized at the same time, unless in an exceptional case where one control mouse developed excess ascites at a much earlier time point than the remaining mice in the group and that single mouse had to be euthanized for humane reasons. Mice usually developed ascites at about day 53 post-tumor cell inoculation or thereafter. The experimental groups which received single treatments generally developed disease at an earlier time point than mice which received the combination treatment. For this reason, varying end points were used in the study for single and combination treatment regimens. A normal B6 mouse was euthanized at the same time as each treatment group. Where present, ascites fluid was immediately recovered from mice using needle and syringe and the ascites volume recorded. This fluid from each mouse was centrifuged for 10 min at 1500 r.p.m. and the upper layer of clear fluid separated from the lower layer of fluid and stored at −20 °C.

### 2.4. Evaluation of Tumor Size and Distribution in Mice

To determine the pattern of tumor spread after in vivo treatments, mice were imaged for RFP (ID8-RFP tumor cells) on the IVIS Spectrum, just after euthanasia and after the removal of ascites. Mice were dissected to expose internal organs or organs were removed, and fluorescence was measured by capturing images on the IVIS Spectrum machine to show differences in RFP spread across organs. Images were used to determine differences in tumor distribution between decitabine and/or selinexor treatment of mice in comparison with control mice. Selected regions of interest (ROIs) were evaluated by Aura Imaging Software (Spectral Instruments Imaging) and the total emission values (fluorescence) in photons/second (photons/s) exported to excel for analysis.

To further determine disease distribution in mice, tissues and organs were fixed in 10% neutral buffered formalin (NBF) overnight. These specimens were then placed in cassettes and embedded in paraffin. Sections (4 µm) were cut from each formalin-fixed paraffin-embedded (FFPE) block and stained with hematoxylin and eosin (H and E). Tumor size and distribution in these organs were studied by pathologist evaluation by scoring the occurrence and size of tumors in tissues using light microscopy and the Aperio Software (Aperio ImageScope by Leica).

### 2.5. Study of Immune Parameters in Treated Mice

To obtain as much data as possible from each mouse, spleens from each mouse were cut into portions for studies, including histology and/or to determine alterations in immune cells or functions with treatments, using monoclonal antibody and flow cytometry as well as other methods. To study the immune cell composition in mouse spleens with treatment, single cell preparations of spleens were stained with fluorescent-labeled monoclonal antibodies for cell surface antigens, including CD45, CD3, CD4, CD8, CD25 (T cells), CD19 (B cells), NK 1.1 (NK cells), F4/80, CD11b, Ly-6G and GR-1 (macrophages) (all from Biolegend, San Diego, CA, USA). Events from multi-color flow cytometry were collected on a BD Fortessa flow cytometer (Becton Dickinson, Franklin Lakes, NJ, USA) and dot plots and histograms analyzed by FloJo analysis (Becton Dickinson) and summarized graphically.

To further decipher changes in anti-tumor potential of mononuclear cell subsets with single and combination treatments of mice, we studied granzyme B secretion in spleen T cells of each treatment group.

To investigate the capacity of T cells to secrete granzyme B (GrB), spleen cells (2 × 10^6^ mononuclear cells/mL) obtained from each mouse were set up in multiple wells in 96-well round-bottomed plates (200 µL/well) in RPMI medium (Corning 10-040-CV) supplemented with 10% fetal bovine serum (FBS; Hyclone Cat. No. SH 30910.03), 1% Penicillin and Streptomycin. Cells were stimulated with purified anti-CD3 antibody (Biolegend Clone 145-2C11: Cat. No. 100340; 5 µg/mL) or cultured with IgG control (Biolegend Cat. No. 400940, or medium) for overnight culture in a 37 °C, 5% humidified CO_2_ incubator. The next day, culture wells from stimulated and unstimulated spleen cell cultures of each mouse were harvested, pooled and centrifuged to collect and freeze the supernatants for analysis of the cytokines and chemokines released. The cells were used to study granzyme B secretion in single cells by intracellular flow cytometry. Briefly, surface phenotyping was performed by monoclonal antibody labeling with antibodies, including anti-CD4, anti-CD8 and anti-CD25 antibodies. Cells were fixed and permeabilized (Cytofast Fix-Perm buffer Cat. No. 421002; Biolegend) and intracellular staining was performed using an anti-granzyme B APC-labeled antibody (Cat. No. 372204; Biolegend) according to the manufacturer’s instructions. Events were acquired on a flow cytometer. Gating was completed in the lymphocyte region and the percentage of CD4+ or CD8+ T cells secreting granzyme B was evaluated using Flojo analysis.

### 2.6. Analysis of Cytokines and Chemokines in Cell Culture Supernatants

Cell culture supernatants from the abovementioned anti-CD3 antibody-stimulated spleen cells were stored at −20 °C and assayed in duplicate in a mouse cytokine 32-Plex Discovery Assay (Eve Technologies, Calgary, Alberta, Canada). These supernatants were also studied for granzyme B using a mouse granzyme B DuoSet ELISA assay (DY 1865-05; R & D Systems).

### 2.7. Statistical Analysis

Data were primarily analyzed using two-sample Student *t* tests (one-tailed or two-tailed or paired as appropriate). The normality assumption was checked using the Shapiro–Wilks test in R. In a few cases where the normality assumption was not satisfied, a nonparametric alternative of the *t* test, the Wilcoxon rank sum test, was used for comparison. In plots, error bars are shown above and below the mean for 1 standard deviation (S.D.). A *p* value of equal to or less than 0.05 was considered statistically significant. Significance levels were indicated as * *p* ≤ 0.05, ** *p* ≤ 0.01, *** *p* ≤ 0.001 and **** *p* ≤ 0.0001.

## 3. Results

### 3.1. Combination Therapy Reduces Ascites Development in Mice

Ovarian cancer was induced in C57BL/6 (B6) mice by inoculation with ID8-RFP cells. In all studies, mice were primed with decitabine (starting at day 6 post-tumor cell inoculation) followed by selinexor administration one dose weekly (Figure 1A). Parallel control groups were set up for comparison of disease outcome with decitabine versus PBS, selinexor versus vehicle, or combination decitabine and selinexor versus PBS and vehicle. Mice were monitored several times weekly, and weights were assessed once each week. In each experiment, at least 5 mice were studied for each treatment group, whereas in some cases, for combination treatment, 10 mice were set up so that additional studies could be performed in this group.

Mice were euthanized when one or more mice in each control group showed severely distended abdomens due to significant ascites accumulation. Contrasting representative panels are shown for abdomens of different mice at the time of euthanasia, including an unmanipulated B6 mouse, combination-treated decitabine and selinexor mice and for PBS and vehicle-treated mice (Figure 1B–D). PBS and vehicle-treated mice had significant abdominal distension due to excess ascites development. In comparison, mice treated with combination decitabine and selinexor were very similar in abdominal size to an unmanipulated B6 mouse (Figure 1B–D). In this study, most mice which received single or combination treatment had flat abdomens as in Figure 1C, and control mice had extended abdomens as in Figure 1D.

**Figure 2:**
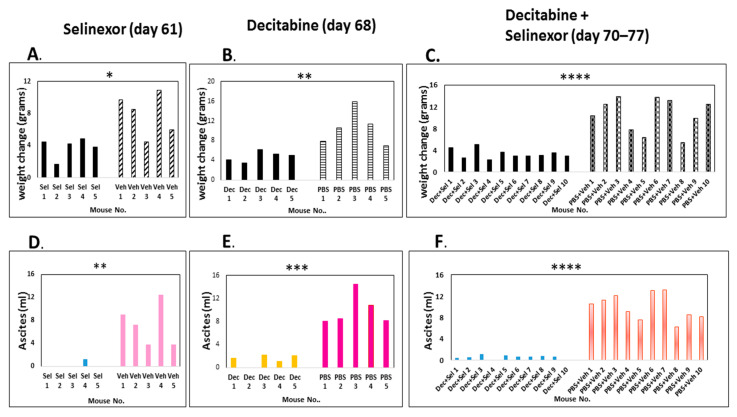
Regulation of weights and ascites development in mice with decitabine and selinexor treatment. Mice were administered ID8-RFP cells, 8 doses of decitabine and 6 doses of selinexor, as described in Figure 1A. Differences in weight as measured at time before any treatments were administered and the day of euthanasia for each treatment group are shown for panels (**A**): selinexor vs. vehicle (*p* ≤ 0.05 *), (**B**): decitabine vs. PBS (*p* ≤ 0.01 **) and (**C**): combination decitabine and selinexor vs. PBS and vehicle (*p* < 0.0001 ****). The volume of ascites recovered from mice is shown in panels (**D**): selinexor vs. vehicle (*p* ≤ 0.01 **), (**E**): decitabine vs. PBS (*p* ≤ 0.001 ***) and (**F**): combination decitabine and selinexor vs. PBS and vehicle (*p* ≤ 0.0001 ****). All statistics were completed with a two-sample *t* test except for (**D**) (selinexor ascites) where the normality assumption was not met, and the analysis was completed using the Wilcoxon rank sum test. Abbreviations: selinexor; sel. decitabine; dec.

Ascites development, a characteristic feature of patients with high-grade serous ovarian cancer (HGSOC), is also a notable feature of the ID8 ovarian cancer mouse model. To determine the effects of experimental treatments on the ovarian cancer outcome, we first studied differences in the gross parameters among groups of mice. The weight increases in the control mice in each group, as measured by the interval before any treatments were administered (day 4) until the point of euthanasia, were plotted. Weights significantly increased in all control groups which received tumor cells in comparison with decitabine and/or selinexor-treated groups (Figure 2A–C).

The weight increase was directly proportional to ascites increase. We recovered a significantly higher volume of hemorrhagic ascites in control mice in comparison with decitabine, selinexor or with combination decitabine and selinexor-treated mice (Figure 2D–F). A summary of the data in the panels for ascites shows that the average and standard deviation over each mouse treatment group were as follows: selinexor 0.26 mL ± 0.58 versus vehicle 7.22 mL ± 3.68 s.d. (*p*≤ 0.01 **, n = 5 mice/group); decitabine 1.4 mL ± 0.90 s.d versus PBS 9.98 mL ± 2.77s.d. (*p* ≤ 0.001 ***, n = 5 mice/group); decitabine and selinexor combination 0.55 mL ± 0.38 s.d. versus PBS and vehicle 9.97 mL ± 2.42 s.d (*p* ≤ 0.0001 ****, n = 10 mice/group). In this experiment, both single and combination treatments significantly decreased ascites development.

**Figure 3:**
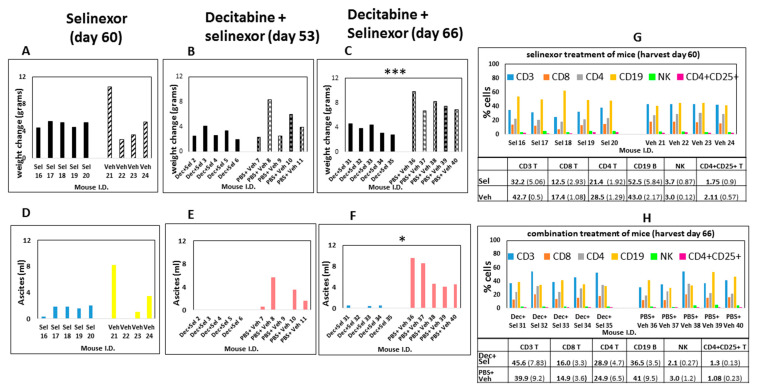
Ascites recovery and phenotypes of cells of treated mice. Mice were administered ID8-RFP cells, 8 doses of decitabine and 5 doses of selinexor. Panels (**A**,**D**) show weight changes and ascites recovery in selinexor-treated mice at the study endpoint day 60. Panels (**B**,**E**) show weight changes and ascites volume recovered in combination decitabine and selinexor-treated mice (day 53). Panels (**C**,**F**) show the same parameters at day 66 euthanasia in combination-treated mice vs. PBS and vehicle controls (*p* ≤ 0.001 *** for weight differences and *p* ≤ 0.05 * for ascites differences). Panel (**G**) shows a summary of flow cytometry analysis of spleen cell phenotypes in selinexor-treated mice (mice I.D. 16–20) and control vehicle mice (mice I.D. 21–24). Panel (**H**) shows cell phenotypes from decitabine and selinexor-treated mice and controls on euthanasia (day 66). Panel (**G**) and panel (**H**) data tables show the average percent cells in flow cytometry dot plots for each cell subset (n = 4 or 5 mice/treatment group as indicated in plots). The standard deviation is shown in brackets for each group.

In another experiment, a similar trend was observed for the combination treatment of mice. A summary of this experiment showed that there was no significant difference in weights or ascites development in mice treated with selinexor in comparison with the vehicle treatment of mice (Figure 3A,D). Additionally, control PBS and vehicle-treated mice developed ascites by day 53 euthanasia (Figure 3E), whereas at this time combination decitabine and selinexor-treated mice had no ascites. There were no significant differences in weight change in combination-treated mice and controls by day 53 (Figure 3B). At day 66 euthanasia, there was a 22-fold lower ascites recovery in combination decitabine and selinexor-treated mice when compared with PBS and vehicle controls (average combination treatment 0.28 mL ± 0.25 s.d. versus control 6.24 mL ± 2.55 s.d.), *p* ≤ 0.05 *, (Figure 3F). There was also a corresponding significant increase in weight in control mice in comparison with decitabine and selinexor-treated mice (Figure 3C). There was also significantly less weight increase (*p* ≤ 0.05 *), and lower ascites formation in decitabine-treated mice in comparison with PBS-treated mice (2.3-fold difference, *p* ≤ 0.05 *), (Supplementary Figure S1A,B). Observations from the data summarized in Figure 2 and Figure 3 indicates that the combination decitabine and selinexor treatment may be more effective in limiting ascites development in mice when compared with selinexor alone.

### 3.2. Decitabine and Selinexor Treatment Inhibits Tumor Growth in Mice

**Figure 4:**
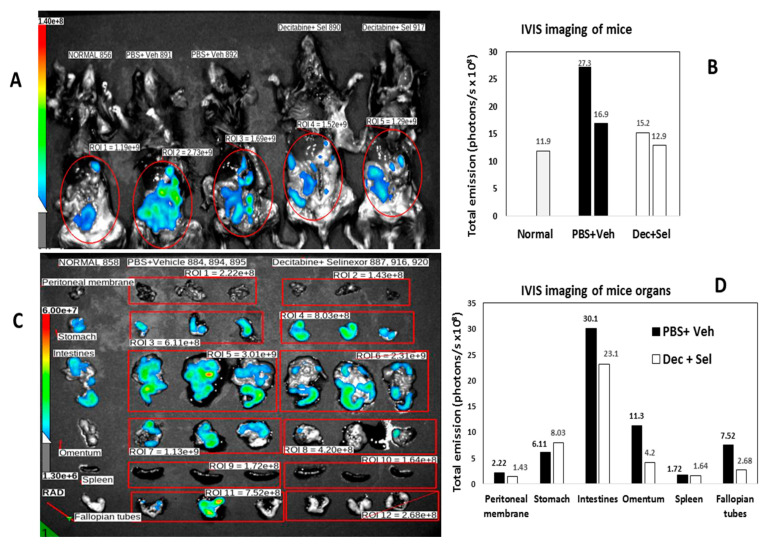

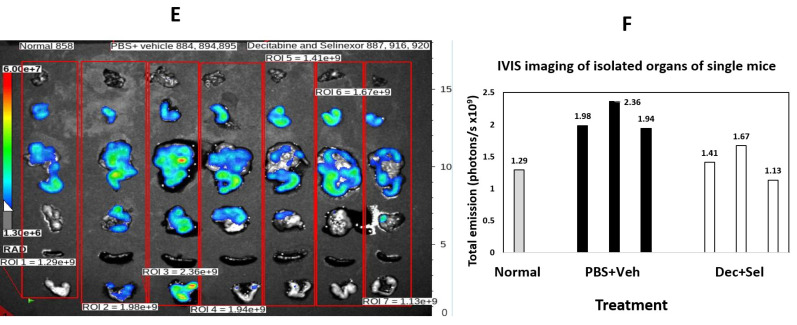
IVIS imaging of mice. In panel (**A**), B6 mice were administered ID8-RFP tumor cells. Mice were treated with 8 doses of decitabine and 6 doses of selinexor (I.D. 890, 917) in combination or with PBS and vehicle (I.D. 891, 892), as shown in Figure 1A. Mice were euthanized at day 74 post-ID8-RFP tumor cell inoculation, abdomens were opened and imaged on an IVIS spectrum. ROIs were selected and total emission (radiance) in photons/s measured. The far-left column shows lowest fluorescence in an unmanipulated B6 mouse. Panel (**B**) is a bar graph plot of total emission in the ROIs of panel (**A**). Panel (**C**) shows imaging of combination-treated mice. B6 mice were administered ID8-RFP tumor cells. Mice (n = 3/group) were treated with decitabine and selinexor combination (I.D. 887, 916, 920) or with PBS and vehicle (I.D. 884, 894, 895). At the time of euthanasia, organs were removed and imaged on an IVIS Spectrum. Regions of interest (ROIs) were selected across the organs of 3 mice of the same treatment together and total emission measured in photons/s where 6.00e+7 = 6 × 10^7^ photons/s. Panels indicate less fluorescence in treated mice than in control mice in intestines, omentum and fallopian tubes. Panel (**D**) is a bar graph plot of Panel (**C**) photons/s measurements. For example, peritoneal membrane of PBS+ vehicle-treated mouse is the value for ROI 1 and fallopian tubes of decitabine + selinexor-treated mice are ROI 12 in the bar graph. Panel (**E**). Mice were treated with ID8-RFP cells followed by decitabine and selinexor (I.D. 887, 916, 920) or with PBS and vehicle (I.D. 884, 894, 895). In Panel (**F**), regions of interest (ROIs) were selected to include organs of single mice as shown (n = 3 mice each treatment group as above). Photons/second values for an unmanipulated B6 mouse are also shown. Panel (**F**) are bar graph plots for ROI 1–7 from panel (**E**), where each bar represents all organs shown for one mouse.

To determine the pattern of tumor spread by IVIS imaging for detection of ID8-RFP-labeled cells, the ROIs of vehicle and PBS-treated mice were compared with the ROIs for combination-treated mice. In decitabine and selinexor-treated mice, there was less fluorescence (total emission in photons/s) than in the ROIs of PBS and vehicle-treated mice (Figure 4A,B). Imaging of different mice (Figure 4C,D) and analysis by treatment group and organs (n = 3), showed that total emission was lower in several organs, including the intestines, omentum, peritoneal membrane and fallopian tubes/ovaries of decitabine and selinexor-treated mice than in PBS and vehicle-treated mice (Figure 4C,D). This suggests that combination treatment with decitabine and selinexor limits tumor growth in several organs of mice. Further analysis of images of mice in Figure 4C shows that individual mice also showed less emission in the combination decitabine and selinexor-treated mice than in the PBS and vehicle-treated mice (Figure 4E,F), indicating the presence of less tumor with combination treatment. Imaging of mice which received selinexor alone showed lower emission than for the control vehicle mice (Supplementary Figure S2A–C), indicating the presence of less tumor in selinexor-treated mice than in vehicle-treated mice. Similar levels of emission were observed in the ROIs of decitabine-treated mice as for PBS-treated mice (Supplementary Figure S2D–F).

### 3.3. Improved Disease Pathology with Decitabine and Selinexor Combination Treatment

**Figure 5:**
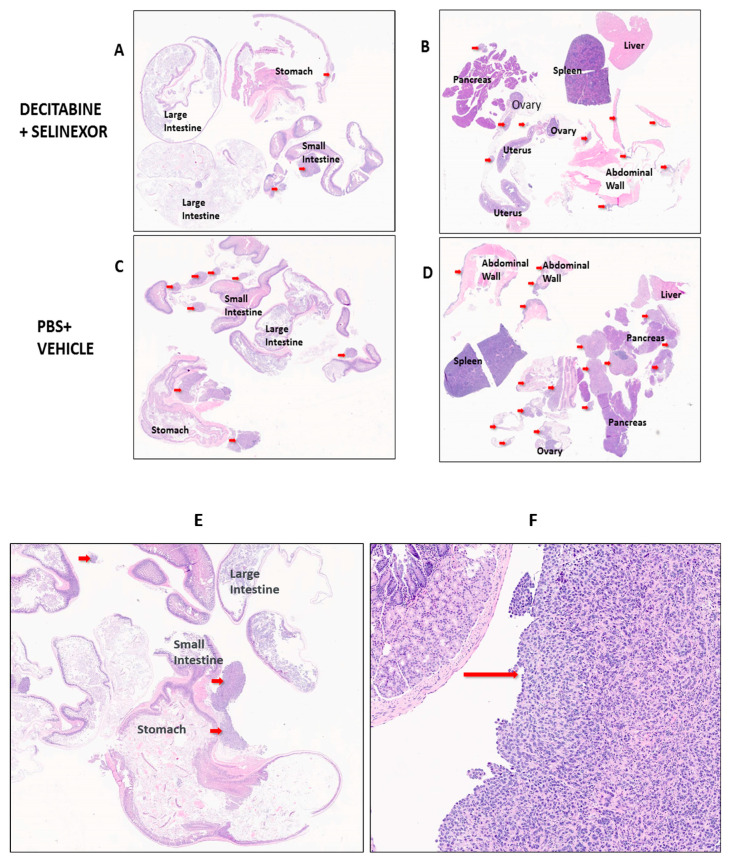
Visualization of ovarian tumor foci in mice. Mice which received tumor cells were treated with 8 doses of decitabine and 6 doses of selinexor (as indicated in Figure 1A). At the time of euthanasia, tissue arrays of each mouse organs were constructed. Arrays were H and E stained and scanned for pathologist evaluation. Arrays shown are representative of tumors (red arrows) on organs of mice for each treatment group. Panels shown are for treatment groups: (**A**,**B**) combination decitabine and selinexor, (**C**,**D**)—PBS and vehicle controls. A similar pattern of tumor spread was found on organs for selinexor and vehicle and for decitabine and PBS mouse groups (Supplementary Figure S3A–H). A summary of these studies is shown in Table 1. Panels (**E**,**F**) show the alimentary tract tissue array with high-power inset of tumor in the small intestine.

To study the size of tumors in mice, we performed a detailed histology on tissue sections of several organs. C57BL/6 mice were administered ID8-RFP tumor cells and treatment agents as described in Figure 1A. Differences in tumor distribution and location between treatment groups of mice were studied in hematoxylin and eosin (H and E)-stained sections of FFPE tissue arrays. Tissue arrays were set up as represented in Figure 5 for pathology evaluation. Tumors in each Figure panel are indicated by red arrows.

In mice treated with PBS and/or vehicle, it was observed that ovarian cancer tumor foci were scattered primarily on the small intestines and peritoneal membranes/abdominal walls with some on ovaries and oviducts and fewer in the large intestines, stomach, pancreas, liver and spleen (Figure 5A–D and Supplementary Figure S3A–H). Findings show that all groups of treated mice had a modest decrease in the number of tumor foci in comparison with the corresponding controls (Table 1). The average number of tumor foci in the mouse arrays was 7.8 tumors in the case of decitabine and selinexor combination-treated mice versus an average of 9.5 tumors in the PBS and decitabine treatment group. This difference was greater than in the selinexor-treated versus vehicle group (av. 6.4 versus 7.0, respectively) or between the decitabine-treated versus PBS group (av. 9.4 vs. 10.6, respectively) (Table 1). Tumor areas were measured as shown in Supplementary Figure S4 using Aperio software. Strikingly, the average surface area for tumor foci was less in single- or combination-treated mice in comparison with controls. These values were selinexor 1.85 mm^2^ versus vehicle 5.52 mm^2^ (*p* ≤ 0.05 *), decitabine 3.91 mm^2^ versus PBS 4.89 mm^2^ (not significant) and decitabine and selinexor combination 2.4 mm^2^ versus controls PBS and vehicle 5.26 mm^2^ (*p* ≤ 0.01 **) (Table 1). These findings provide evidence that there is significantly improved tumor pathology (less disease) in mice treated with single treatment selinexor or with combination decitabine and selinexor in comparison with their corresponding controls.

**Table 1:** Measurements of tumor foci in mice with ovarian cancer *.

Treatment	Average Tumor Size (mm^2^)	Average Tumors/Mouse
Selinexor	1.85	6.4
Vehicle control	5.52	7
Decitabine	3.91	9.4
PBS control	4.89	10.6
Decitabine + Selinexor	2.4	7.8
PBS + Vehicle control	5.26	9.5

* Mice were inoculated with ID8-RFP tumor cells and administered treatments of eight doses of decitabine and six doses of selinexor as described (Figure 1A). Portions of organs were FFPE-embedded in two blocks and two tissue arrays made for H and E staining for each mouse. The stained slides were scanned as shown in Figure 5, and all the measurable tumor foci in the arrays were counted for mice in each treatment group. Data shown are for the pathology evaluation of tissue sections for single treatment groups (10 arrays for 5 mice/treatment) and combination treatments groups (20 arrays for 10 mice/treatment). Data shown in Table 1 are for observations made for 40 mice in total, in one of two representative experiments.

A summary of the number of tumors in both arrays for each mouse was made. The size (area in mm^2^) of each tumor was measured. From this, the total surface area of all tumors in mice from each treatment group was summarized. The average tumor size was determined as the total surface area of all tumors in mice from each treatment group/the total number of tumors in that treatment group.

A two-sample *t* test was performed to compare the average tumor sizes in mice across corresponding treatment groups. There was a significant difference in the average tumor size in selinexor vs. vehicle groups (n = 5 mice/group, *p*-value ≤ 0.05 *). There was a significant difference in the average tumor size in combination decitabine + selinexor vs. PBS + vehicle groups (n = 10 mice/group, *p*-value ≤ 0.01 **). There was no significant difference in the average tumor size in decitabine vs. PBS group.

### 3.4. Regulation of Immune Parameters with Combination Treatment of Mice

**Figure 6:**
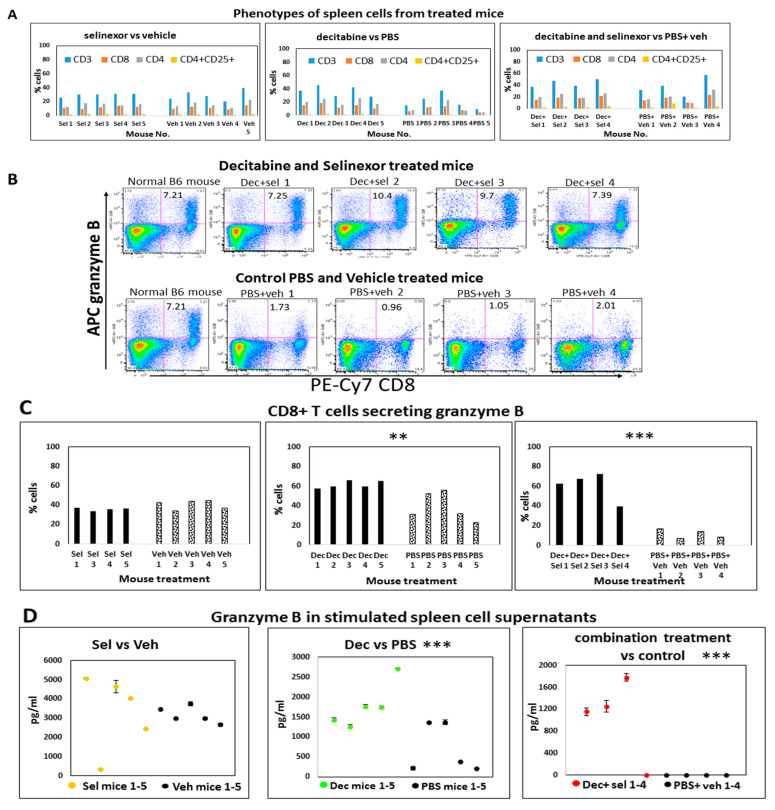
Phenotypes and function in single- and combination-treated mice. Mice were administered ID8-RFP cells and treated with 8 doses of decitabine and 6 doses of selinexor, as described in Figure 1A. At the time of euthanasia, spleens were removed and single-cell suspensions were prepared. Unstimulated cells from each treatment group were monoclonal antibody-stained and had multi-color flow cytometry performed. Events were acquired on a flow cytometer and the analysis was performed using FloJo. Percentages shown in bar graphs in panel (**A**) are a summary of dot plot analysis using flow cytometry for each mouse. Panel (**B**). Some spleen cells were stimulated with anti-CD3 antibody overnight. Cells and supernatants were recovered by centrifugation the next day. Cells were monoclonal antibody-labeled for CD4, CD8, CD25 and other surface markers. They were fixed and permeabilized, stained for granzyme B, events acquired on a flow cytometer and dot plot readouts plotted. Panel (**B**) shows granzyme B secretion by CD8+ T cells of combination-treated mice in upper right quadrants. Unstimulated CD4+ or CD8+ T cells or stimulated CD4+ T cells secreted about 1% granzyme B. Panel (**C**) summarizes the fraction of stimulated CD8 T cells which secrete granzyme B in each treatment group. Bars represent (%CD8+GrB+ T cells in upper right quadrants of dot plots)/total %CD8 T cells ×100). Data from each mouse are represented by one bar (n = 4 or 5 mice). Results show significant differences in granzyme B-secreting CD8+ T cells for decitabine vs. PBS (*p* ≤ 0.01 **) and decitabine and selinexor vs. PBS and vehicle(*p* ≤ 0.001 ***) groups. Panel (**D**) shows granzyme B levels (pg/mL) released in anti-CD3 antibody-stimulated spleen cell cultures. Supernatants were assayed in duplicate wells by ELISA. Data for each mouse are represented by one circle. The mean granzyme B concentration over the 2 wells and standard deviation are shown for each mouse. Results show significant differences between granzyme B release for decitabine vs. PBS (*p* ≤ 0.001 ***) and decitabine and selinexor vs. PBS and vehicle (*p* ≤ 0.001 ***) groups. Trends of granzyme B in supernatants are representative of 2 experiments. The analysis in Figure 6 was completed using a two-sample Student *t* test.

Mice were treated with decitabine and/or selinexor as described (Figure 1A). At the study end point, portions of individual spleens were used to prepare single-cell suspensions to study how immune cell compositions in mice were regulated with treatment. Using these unstimulated spleen cells, we investigated changes in T cells, B cells, NK cells, macrophages (F4/80, CD11b, Ly-6G, GR-1) and the CD4+CD25+ T regulatory cell population in all groups of mice using monoclonal antibody labeling of spleen cells and flow cytometry. We did not observe any changes in macrophage subsets between the control and treated groups. Results revealed that there were modest increases in unstimulated CD3, CD4 and CD8 T cells obtained from combination decitabine and selinexor-treated mice and from decitabine-treated mice in comparison with their corresponding controls (Figure 3H and Figure 6A). We noticed a modest decrease in T cell subsets in selinexor- versus vehicle-treated mice (Figure 3G). Overall, there is a trend towards an increase in the percentage of CD3, CD4 and CD8 T cells in mice treated with decitabine and selinexor or with decitabine alone but not with selinexor treatment alone.

### 3.5. Higher Levels of Granzyme B Are Secreted in the CD8-Positive T Cells of Combination-Treated Mice

Granzyme B is primarily a CD8+ T cell (and NK cell) cytotoxic molecule effective against tumor cells [42]. To decipher changes in this parameter in treated mice, we investigated granzyme B secretion in T cells using flow cytometry and granzyme B released in cell culture supernatant using ELISA.

We next studied granzyme B secretion in spleens in situ using IHC for these same selinexor-treated and vehicle-treated mice. In IHC stained sections, granzyme B was localized in the lymphocytes of the red pulp of spleens with very low occurrence (1–2 per high-power field). There were no significant differences with granzyme B in tissue sections between treated and control mice.

Spleen cells from mice which received decitabine and selinexor had on average 8.7% (±1.60 s.d.) granzyme B-secreting CD8+ T cells in dot plots, whereas the PBS and vehicle-treated mice had on average 1.4% (s.d. ± 0.51, n = 4 mice) granzyme B-secreting CD8+ T cells (a six-fold difference), after stimulation with anti-CD3 antibody (Figure 6B). A study of the portion of CD8+ T cells obtained in spleen cell cultures which expressed granzyme B (normalized to total CD8+ stimulated T cells) showed that there was a significant increase in this parameter between cells obtained from decitabine and selinexor combination-treated mice (average 55.1%, s.d. 13.95 versus 10.5%, s.d. 4.13, n = 4; *p* ≤ 0.001 ***). We also observed an increase in granzyme B-secreting CD8+ T cells (average 61.5%, s.d. 3.78, n = 5) with decitabine treatment of mice in comparison with PBS-treated mice (39.0%, s.d. ±41.5, n = 5, *p* ≤ 0.01 **). For selinexor-treated mice, we did not observe a significant difference in granzyme B secretion in CD8+ T cells (selinexor average 35.8%, n = 4 vs. vehicle 40.6%, n = 5) (Figure 6C).

Testing of the supernatants from the anti-CD3 antibody-stimulated spleen cells showed a significant increase in granzyme B in cell culture supernatants obtained from decitabine-treated mice and from combination decitabine and selinexor-treated mice in comparison with controls, whereas this increase in granzyme B in culture supernatants was not noticed after selinexor treatment of mice (Figure 6D). A similar outcome was observed in another experiment.

To further investigate T cell responses from mice with single and combination treatments, we studied changes in CD8+CD25+-activated T cells present in the total percent of CD8 T cells in anti-CD3 antibody-stimulated spleen cell cultures. There was a modest though not significant increase in the fraction of CD8+CD25+-activated CD8 T cells from combination-treated decitabine and selinexor-treated mice in comparison with their controls and for decitabine-treated mice versus PBS-treated mice.

Taken together, the decitabine and selinexor combination treatment induced CD8+ T cells with a greater capacity to secrete granzyme B than the corresponding controls. A similar pattern was noticed for decitabine-alone treatment in comparison with PBS. Selinexor-alone treatment of mice did not induce T cells with a greater capacity to secrete granzyme B than corresponding controls.

### 3.6. Combination Treatment of Mice Enhances the Secretion of Cytokines

To further decipher changes in the immune outcomes among mice in each treatment group, we studied the supernatants obtained from the cultures of anti-CD3 antibody stimulated spleen cells which were stored from the abovementioned studies. Analyses of supernatants were conducted on a mouse cytokine 32-Plex Discovery Assay platform. The plots show the mean and standard deviation over duplicate wells for each cytokine or chemokine reported. The results represent the 10 most predominantly expressed soluble molecules released in culture supernatants. Additional soluble molecules secreted in lower amounts (less than 150 pg/mL) include IL-3, RANTES, IL-10 and TNF-α. The unstimulated spleen cells released negligible amounts of soluble molecules in culture.

**Figure 7:**
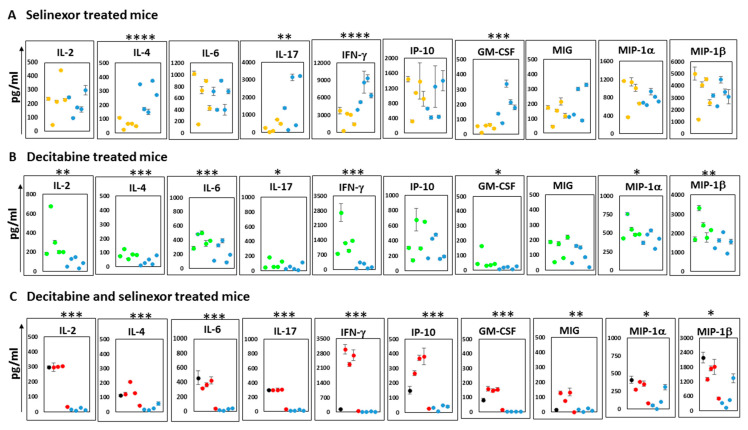
Soluble molecules secreted from spleens of treated mice. ID8-RFP cells and treated with 8 doses of decitabine and 6 doses of selinexor, as described in Figure 1A. Anti-CD3 antibody-stimulated spleen cell supernatants of different mouse groups were studied in a 32-Plex cytokine array panel (Eve Technologies). Data are shown for cytokine/chemokine secretion in supernatants obtained from the spleen cells of mice groups treated as indicated for: Panel (**A**) selinexor and control vehicle, Panel (**B**) decitabine and control PBS, and Panel (**C**) combination decitabine and selinexor and control PBS and vehicle. Data for control mice are shown in blue dots in each panel. The black circle in panel C is for a normal B6 mouse. Each data point represents the average soluble molecule amount (pg/mL) released in culture for each mouse supernatant assayed in duplicate. Data are shown for 4–5 mice per group. Plots show mean ± 1 s.d. and significance levels * *p* ≤ 0.05, ** *p* ≤ 0.01, *** *p* ≤ 0.001 and **** *p* ≤ 0.0001.

A study of supernatants from mice spleen cells stimulated with anti-CD3 antibody revealed unique differences in the patterns of cytokine release. With supernatants from the selinexor treatment of mice, there was significantly less IL-4, IL-17, IFN-γ and GM-CSF than that obtained from mice treated with the vehicle (Figure 7A). In contrast, cultures from the stimulated spleen cells of mice treated with decitabine showed higher levels of IL-2, IL-4, IL-6, IL-17, IFN-γ, GM-CSF, MIP-1α and MIP-1β than mice treated with the PBS (Figure 7B). Cells obtained from mice which received combination treatment decitabine and selinexor were capable of secreting significantly higher levels of all 10 cytokines/chemokines studied in Figure 7C. The soluble molecules secreted in the highest amounts in the cell culture supernatants of combination-treated mice were IFN-γ and MIP-1β. The relevance of levels of specific soluble molecules in ovarian cancer outcomes is controversial. However, our observations suggest that decitabine and selinexor used in a combination treatment of mice can prime cells to secrete a broader program of cytokines and chemokines (both T cell-activating and macrophage-activating) on ex vivo stimulation than either decitabine or selinexor used as standalone treatment. This raises the possibility that combination treatment in mice can induce mice to a greater level of immune activation than a single treatment.

## 4. Discussion

Anti-cancer drugs, such as decitabine and selinexor, are FDA approved for use in some hematologic malignancies [19,20,33]. Most reports have primarily focused on the impact of decitabine and selinexor on cancer cells, but more recently these agents have been in clinical trials in a wider range of cancers due to their anti-tumor immune modulatory properties. Our investigations evaluated a combination of decitabine and selinexor in a mouse model of ovarian cancer.

In most of these experiments, mice were euthanized after day 60 post-tumor cell inoculation, a time at which the control mice in each tumor arm had significant ascites and had therefore reached the time for humane euthanasia. However, this study had different euthanasia end points in each experimental arm. This is possibly because the mice in different groups received different amounts of treatment manipulations, which may have led to the development of disease/ascites at a different rate. Therefore, mice which had six doses of vehicle had the earliest end point (day 61), mice receiving eight doses of PBS had the second earliest end point (day 68) and mice which had 14 treatments (PBS and vehicle) were humanely euthanized later, that is, after day 70 of tumor cell inoculation.

In this study, mice which received a combination treatment had significantly reduced tumor size and significantly lower ascites development than corresponding control mice. We investigated tumor spread using histology and the IVIS imaging platform. The IVIS imaging of mice was performed after removal of ascites fluid because the increased presence of ascites in the abdomens of control mice could prevent accurate imaging in the abdominal area. Capturing images in the IVIS machine chamber may also be influenced by several other extraneous factors. These include how many mice are placed in the chamber at the same time, the location of mice in the chamber, whether there is undetectable moisture on the imaging surface and whether there is minimal/uneven dehydration in the ROI areas of mice. In these studies, we made all attempts to keep these factors as consistent as possible for imaging of treatment and control groups to limit the extent to which these conditions could alter our results. Studies showed that there was less fluorescence (RFP) detected in combination decitabine and selinexor-treated mice in comparison with PBS and vehicle-treated mice. By pathology evaluation there was a trend towards having fewer tumors in combination-treated mice in comparison with controls, and it was found that the average tumor size in the combination-treated decitabine and selinexor-treated mice was significantly less than in the corresponding control mice (Table 1). Thus, by both IVIS imaging and by histological evaluation, there was some disease improvement observed with combination treatment.

Selinexor treatment of mice also resulted in a significantly smaller average tumor size in mice than in vehicle-treated mice as determined by pathological evaluation. It is noteworthy that with IVIS imaging there was also lower fluorescence in selinexor-treated mice than in control vehicle mice. In the case of decitabine and control PBS, however, through both IVIS imaging and histology, we did not observe any differences in disease improvement. However, decitabine used alone in mice significantly limited ascites development, and thus this treatment may yet be of limited benefit against ovarian cancer.

In our cytokine array study (Figure 7A) of data obtained from selinexor-treated mice, we observed that there was significantly lower IFN-γ released in cultures of anti-CD3 antibody-stimulated spleens than in the vehicle control spleens. Selinexor is a selective inhibitor of nuclear export. It retains all XPO1 target proteins in the nucleus [43,44], including STAT-1 and STAT-3, both transcription factors which are important in the normal functioning of the immune system. The inhibition of the nuclear export of STAT-1 can reduce the capacity of cells to upregulate IFN-γ-responsive genes and, consequently, IFN-γ secretion [45,46]. It is plausible that in our system the tendencies of lower cytokine and chemokine secretions from selinexor-treated mice than from vehicle-treated mice are due to the activity of selinexor trapping several proteins needed for the secretion of these soluble molecules in the nucleus. In comparison with the corresponding controls, the effect of low IFN-γ secretion in spleen cell cultures appeared to be overcome in cultures of mice treated with decitabine or with decitabine and selinexor, where the reverse pattern of higher IFN-γ in cultures of treated mice than in corresponding controls is observed.

Others report that strong immunomodulatory effects have been identified with the use of decitabine. These include increased T cell infiltration in the tumor and priming of the tumor for treatment of agents, such as immune checkpoint blockade therapy. In our model, in comparison with the PBS control, decitabine treatment alone resulted in improved CD8+ T cell responses both for granzyme B secretion in CD8+ T cells using flow cytometry and for the release of several soluble molecules, including significantly higher levels of IFN-γ in culture. In comparison with corresponding PBS-treated mice, cultures from decitabine-treated mice were rich in 8 of the 10 cytokines studied in detail, including IL-2, IFN-γ, MIP-1α and MIP-1β. Thus, we see that decitabine has the capacity to boost immune responses in our model of ovarian cancer. Others also showed that CD8+ T cells from systemic decitabine-treated mice expressed upregulation of granzyme B and IFN-γ [26]. Additionally, it is reported that IFN-γ T cells may contribute to clinical responses after decitabine treatment [47].

A combination of decitabine and selinexor effectively limits ovarian cancer and potently enhances anti-tumor immune responses. Notably, after in vitro stimulation, granzyme B in CD8+ T cells is highly secreted, and a wide spectrum of cytokines and chemokines which may be relevant to the normal functioning of the immune system are released in culture. Interestingly, IL-2, which promotes the differentiation of effector T cells, IFN-γ and interferon gamma-induced protein (IP-10), are among some of the soluble molecules secreted by spleen cells of decitabine and selinexor-treated mice. IP-10 (CXCL-10) attracts T cells, NK cells, monocytes, macrophages and dendritic cells and prevents angiogenesis. The potential of this chemokine to be increased in the TME of decitabine and selinexor-treated mice above control mice may contribute to increased Th1 lymphocytes in the TME of mice with this combination treatment. MIP-1α (CCL-3) and MIP-1β (CCL-4), which are secreted by stimulated macrophages, were also secreted in cultures from cells of combination-treated mice in significantly higher levels than controls. Whereas combination treatment induced several macrophage and T cell anti-tumor cytokines, this pattern was lacking in cells from selinexor-alone-treated mice, which may indicate that selinexor treatment of mice does not have as great a capacity to induce anti-tumor responses as the combination decitabine and selinexor treatment of mice. The potential of cytokines and chemokines to be secreted by mice treated with combination decitabine and selinexor treatment above corresponding controls may be of great anti-tumor benefit in ovarian cancer for several reasons. Interestingly, as mentioned previously, many of these soluble molecules regulate T cell anti-tumor immunity or can attract T cells into the TME. The ovarian TME consists of low levels of intra-tumoral T lymphocytes (cold tumor) [48]. Several reports indicate that the increased presence of immunocompetent T lymphocytes in the TME is associated with better prognosis in several cancers, including ovarian cancer [49]. Therapy combinations, such as decitabine and selinexor which cause the release of soluble molecules which can boost T cell presence or function in the TME, may provide a strategy for an improved response to therapy.

## 5. Conclusions

Our study evaluated the efficacy of single treatment or combination decitabine and selinexor treatment of mice by investigating disease pathology and immune response parameters. Single treatment selinexor as well as combination treatment decitabine and selinexor significantly limited the size of tumors in mice. However, the outcome of combination treatment decitabine and selinexor was accompanied by an increase in granzyme B secretion in CD8+ T cells and by a significant release of all the macrophage and T cell cytokines studied, in comparison with observations made for the PBS and vehicle control treatments. These potent anti-tumor responses were not observed with selinexor vs. vehicle when used as a single treatment in mice. Agents such as a decitabine and selinexor combination which induce disease improvement accompanied by improved anti-tumor immune responses hold promise for ovarian cancer, a disease which has a paucity of functional immunocompetent T cells in the tumor microenvironment, as these agents may boost T cell immunity.

Due to the low survival in ovarian cancer patients, novel clinical trials are urgently needed to manage this disease. Our findings set the stage for clinical trials in ovarian cancer with decitabine and selinexor used in combination with or without other treatment agents.

## Data Availability

Not applicable.

## Supplementary Materials

The following supporting information can be downloaded at: https://www.mdpi.com/article/10.3390/cancers15184541/s1, Figure S1: Ascites recovery and weight changes in decitabine-treated mice. B6 mice were administered ID8-RFP tumor cells and decitabine as in Figure 1A. On euthanasia, Panel S1A shows a significant increase in weight in PBS- treated mice above decitabine-treated mice (*p* ≤ 0.05 *). Panel S1B shows significantly less ascites in decitabine-treated mice in comparison with control PBS-treated mice (*p* ≤ 0.05 *); Figure S2: IVIS imaging of single treatment mice. In panel S2A, B6 mice were administered ID8-RFP tumor cells, followed by selinexor treatment as shown in Figure 1A. Mice were euthanized at day 61 post-tumor cell injection. Images of mice with abdominal areas exposed show measurements of total emission (photons/s) in ROIs. Images for control vehicle are shown in panel S2B, and Panel S2C shows a bar graph of the emission values (photons/s) in Panels S2A and S2B. Similarly, Panels S2D and S2E are for imaging of decitabine-treated and PBS control mice. Plot S2F is a summary of panels S2D and S2E ROI values; Figure S3: Ovarian tumor foci in single treatment mice. Mice (B6) were administered ID8-RFP cells at day 0, followed by selinexor or decitabine in a single regimen. Tissue arrays of H and E stained organs show tumor distribution (red arrows) for FFPE sections of mice treated with (S3A,B) selinexor, (S3C,D) vehicle, (S3E,F) decitabine and (S3G,H) PBS. A summary of these studies is shown in Table 1; Figure S4: Measurement of tumor size: tumors in scanned images as shown in Figure 5 were measured using the Aperio Software (Aperio ImageScope by Leica). Data obtained from measurements of all tumors in mice groups were evaluated and summarized in Table 1.
