# Supplementary material for: Selinexor in Combination with Decitabine Attenuates Ovarian Cancer in Mice
Source: Cancers (Basel). 2023 Sep 13;15(18):4541. doi: 10.3390/cancers15184541 (PMC10526280; doi:10.3390/cancers15184541)

S1A

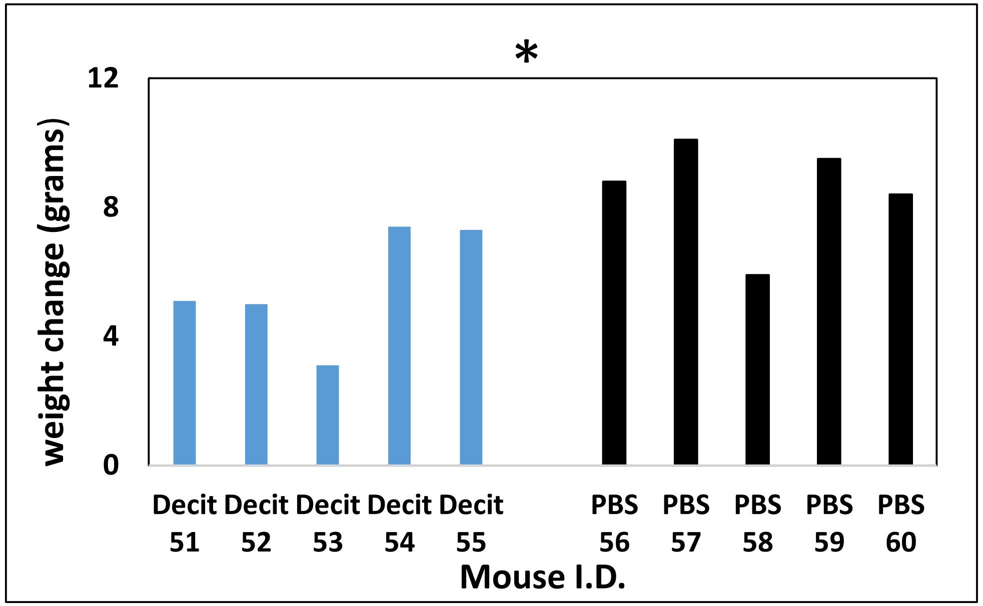
*
12
8
4
0
weight change (grams)
Decit 51
Decit 52
Decit 53
Decit 54
Decit 55
PBS 56
PBS 57
PBS 58
PBS 59
PBS 60
Mouse I.D.

S1B

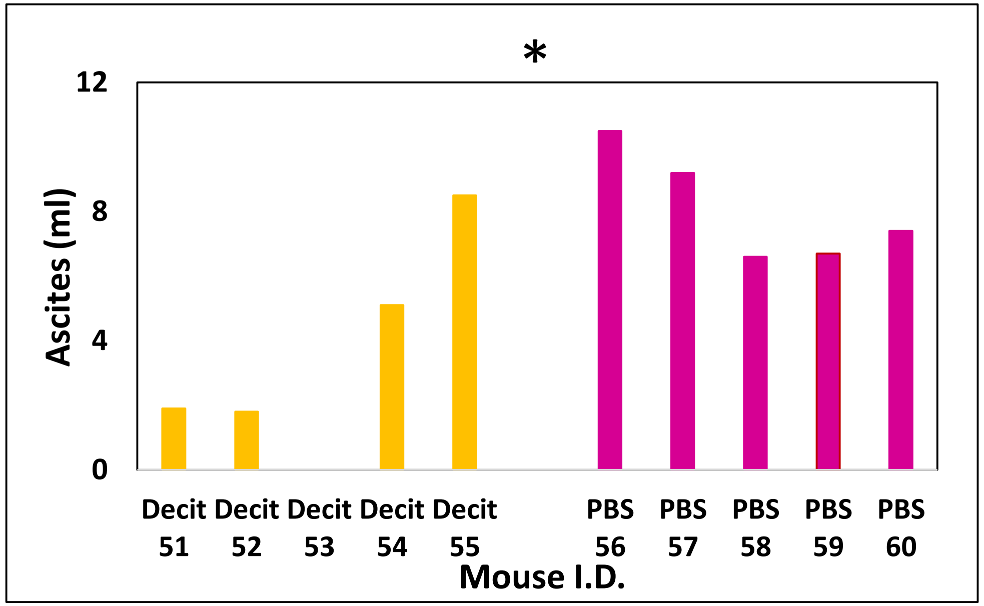
*
12
8
4
0
Ascites (ml)
Decit 51
Decit 52
Decit 53
Decit 54
Decit 55
PBS 56
PBS 57
PBS 58
PBS 59
PBS 60
Mouse I.D.

S2A
Selinexor

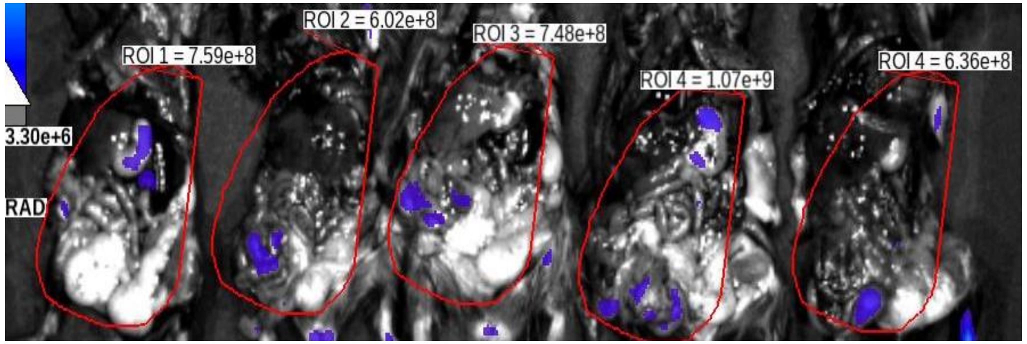

S2B
Vehicle

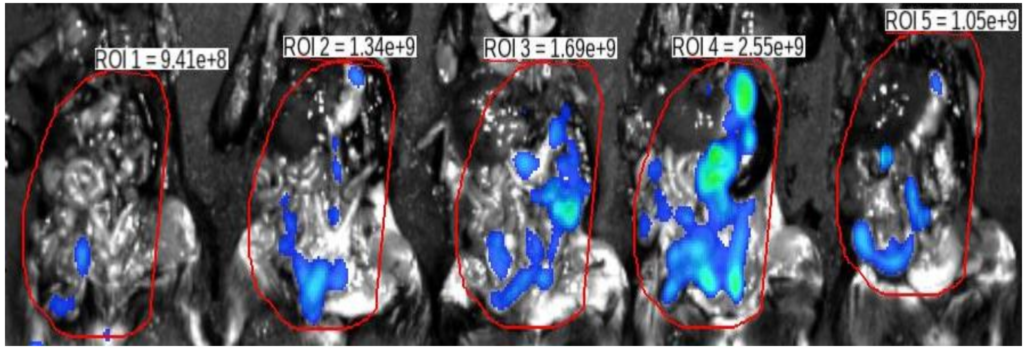

S2C

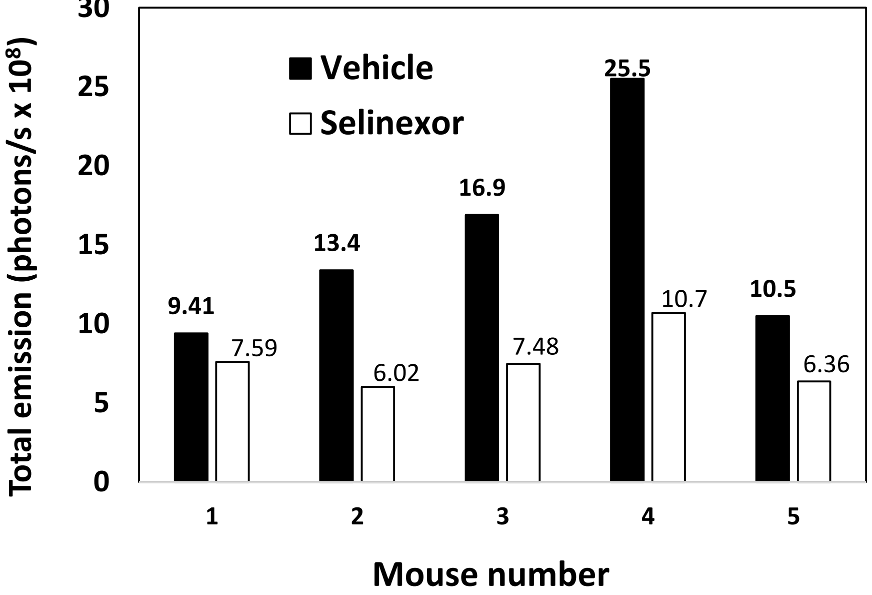

S2D
Decitabine

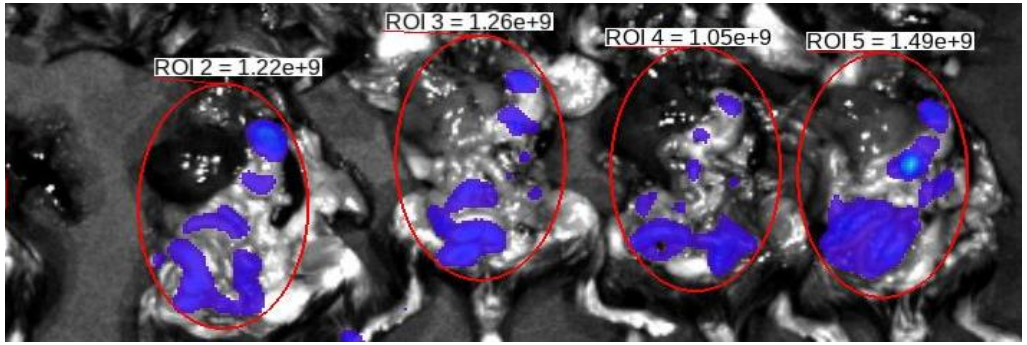

S2E
PBS

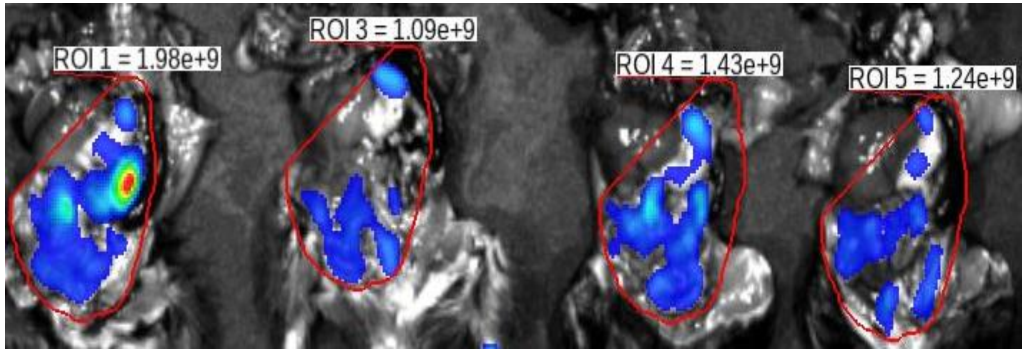

S2F

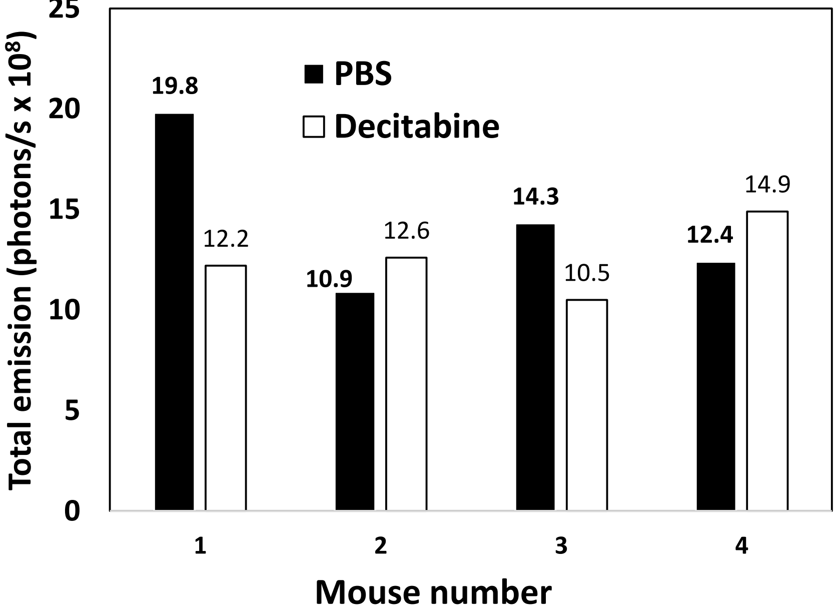

SELINEXOR

S3A

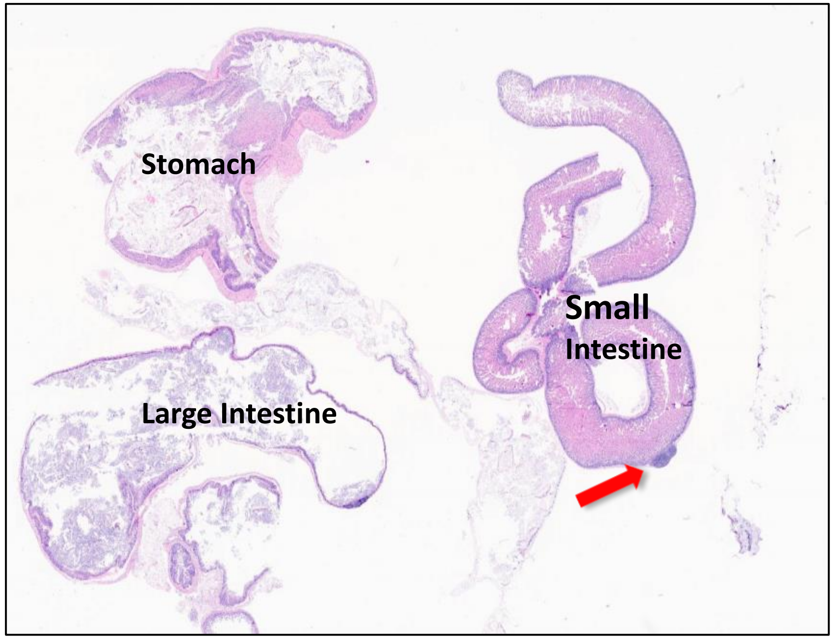

S3B

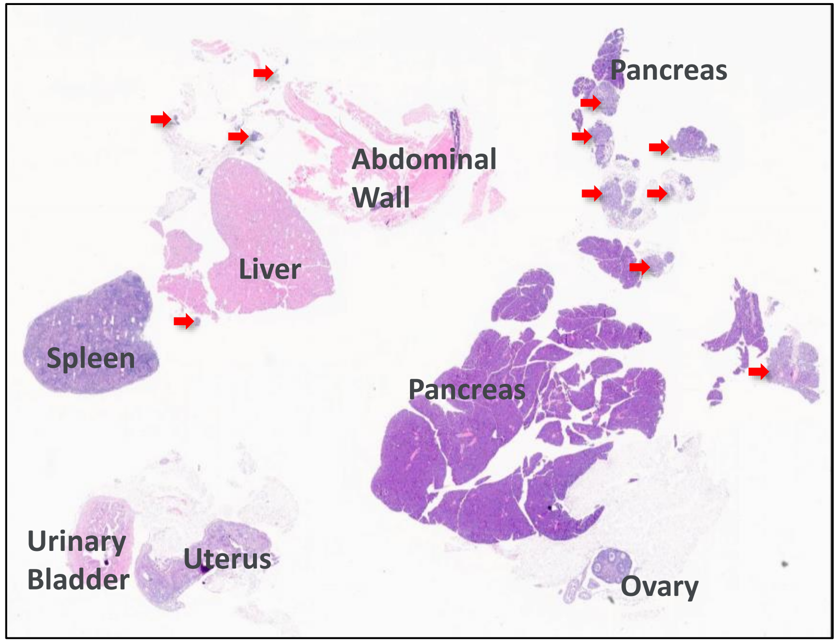

VEHICLE

S3C

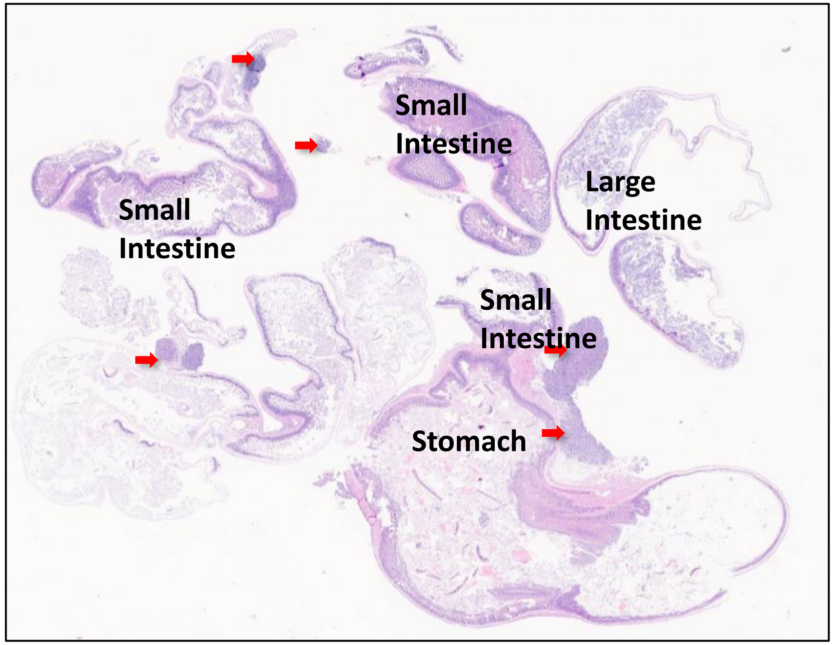

S3D

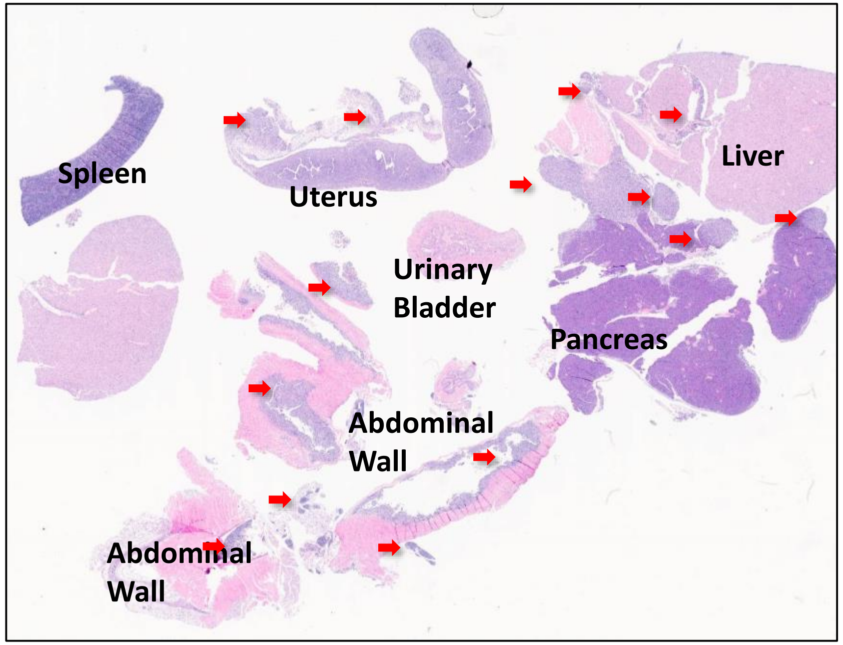

DECITABINE

S3E

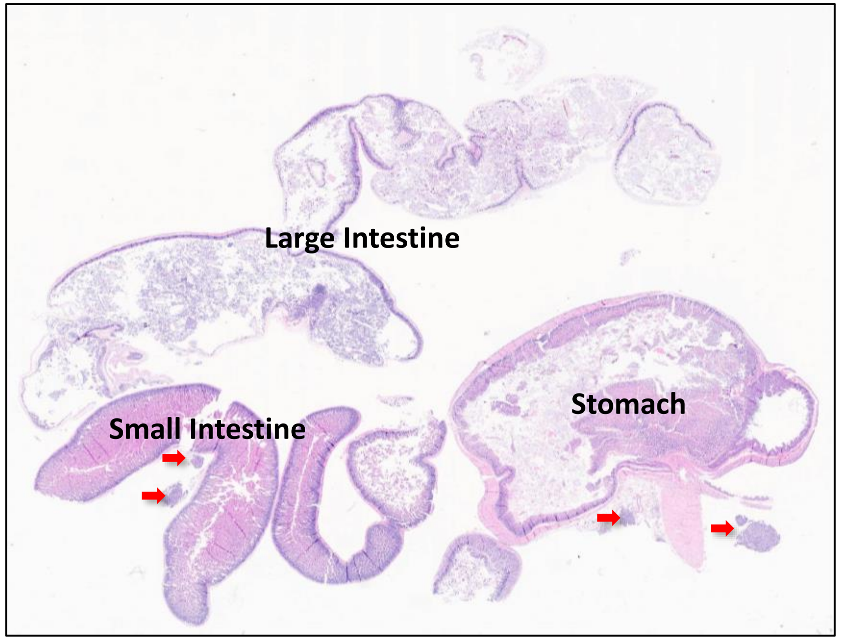

S3F

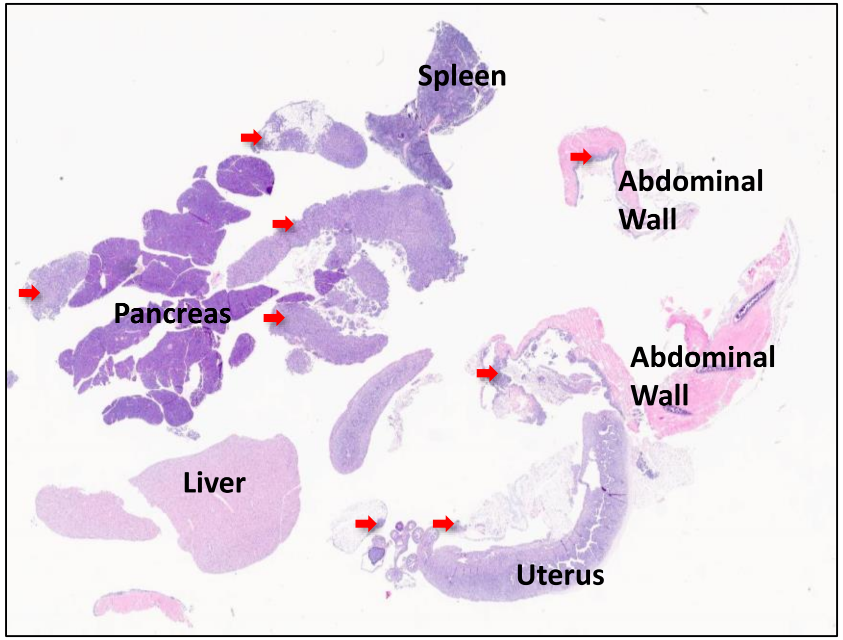

PBS

S3G

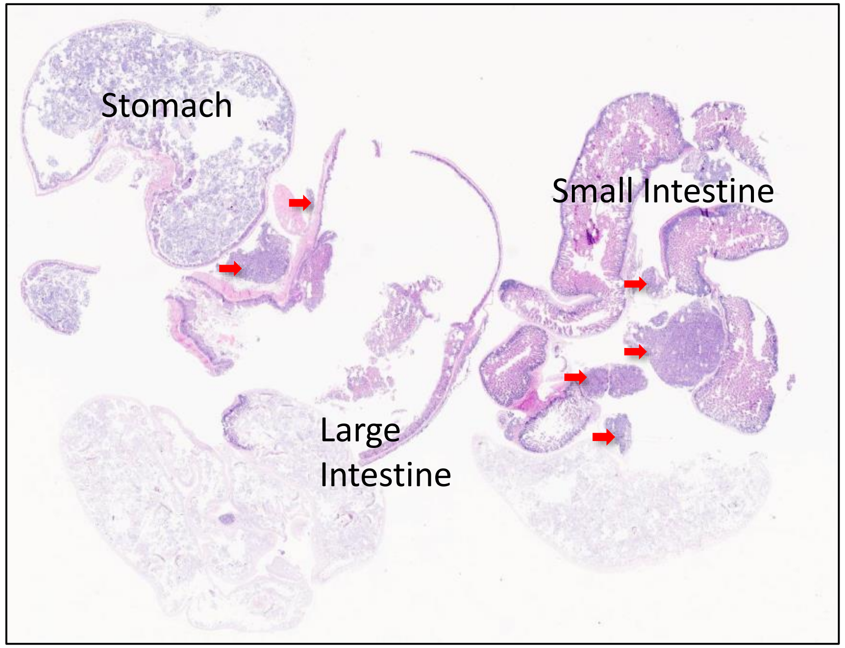

S3H

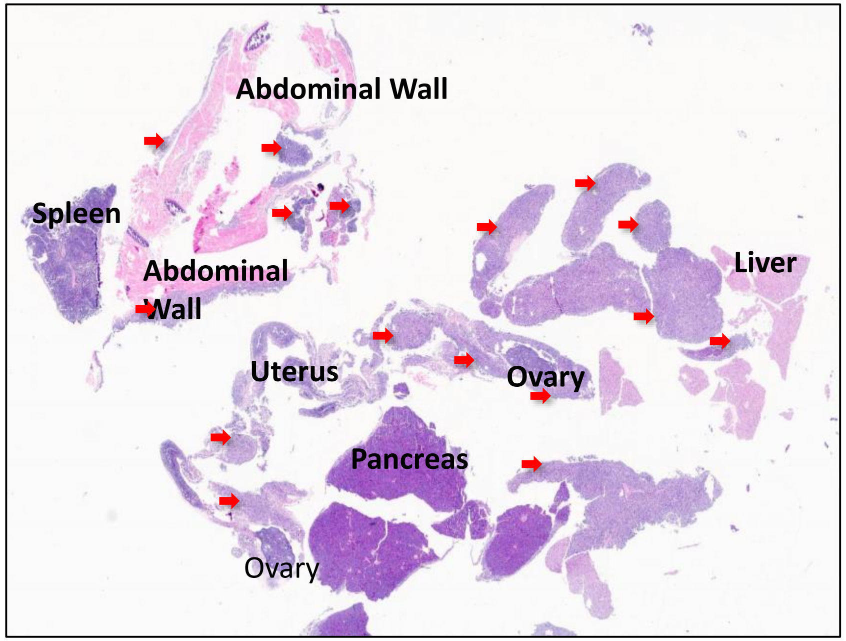

S4

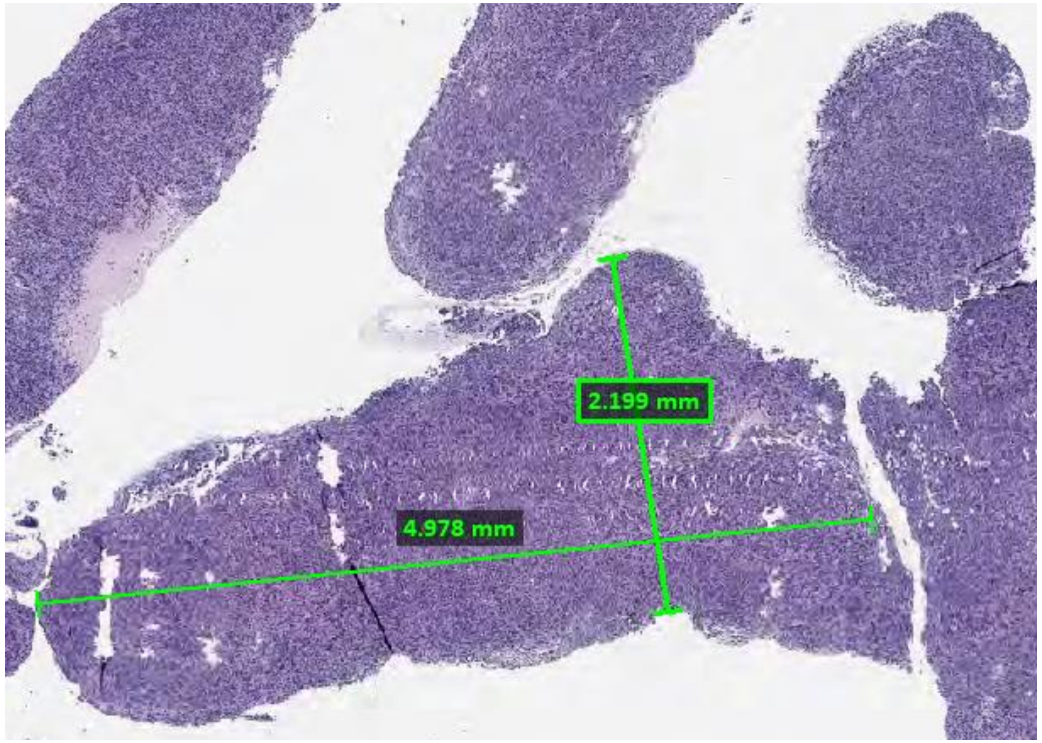

Supplement: Supplementary file 1 [file cancers-15-04541-s001.zip › cancers-2583627-supplementary.pdf]
